# Creating Excess Electrons at the Anatase TiO_2_(101) Surface

**DOI:** 10.1007/s11244-016-0706-8

**Published:** 2016-09-07

**Authors:** D. T. Payne, Y. Zhang, C. L. Pang, H. H. Fielding, G. Thornton

**Affiliations:** 1 Department of Chemistry, University College London, London, WC1H 0AJ UK; 2 London Centre for Nanotechnology, University College London, London, WC1H 0AH UK

**Keywords:** TiO_2_, Anatase, Defect creation, Hydroxyl, Water, Excess electrons

## Abstract

Excess electrons facilitate redox reactions at the technologically relevant anatase TiO_2_(101) surface. The availability of these electrons is related to the defect concentration at the surface. We present two-photon (2PPE, 3.10–3.54 eV) and ultraviolet (UPS, 21.2 & 40.8 eV) photoemission spectroscopy measurements evidencing an increased concentration of excess electrons following electron bombardment at room temperature. Irradiation-induced surface oxygen vacancies are known to migrate into the sub-surface at this temperature, quickly equilibrating the surface defect concentration. Hence, we propose that the irradiated surface is hydroxylated. Peaks in UPS difference spectra are observed centred 8.45, 6.50 and 0.73 eV below the Fermi level, which are associated with the 3σ and 1π hydroxyl molecular orbitals and Ti 3d band gap states, respectively. The higher concentration of excess electrons at the hydroxylated anatase (101) surface may increase the potential for redox reactions.

## Introduction

Titanium dioxide (TiO_2_) is a prototypical material used in numerous and varied applications, including photocatalysis [1, 2]. Of the two main TiO_2_ polymorphs relevant to industry, rutile and anatase, the former has received the most scientific attention. However, anatase is believed to be the more catalytically active phase [3] and is therefore preferred in most industrial applications. Anatase is a metastable phase of TiO_2_ and converts to the more thermodynamically stable rutile phase at temperatures above ~1020 K for relatively large single crystal samples [4]. The most stable anatase crystal facet is the (101) termination, which forms over 94 % of the surface for macroscopic crystals [5, 6]. Hence, it is the (101) surface that forms the majority fraction of catalytic interfaces. Interestingly, theoretical and experimental studies suggest that the minority (001) surface may be catalytically more reactive [5, 7, 8], although the relative catalytic activity of anatase surfaces remains controversial [9].

**Fig. 1:**
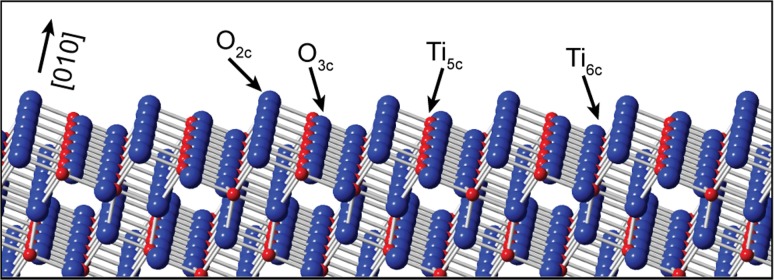
Ball and stick model of the anatase TiO_2_(101) surface. The surface comprises two- and three-fold coordinated O atoms (*blue*) and five- and six-fold coordinated Ti atoms (*red*)

The anatase (101) surface has previously been investigated by low-energy electron diffraction (LEED) [10] and scanning probe microscopy [4, 11–20] as well as photoemission [21–26], infrared reflection absorption [13] and near edge X-ray absorption fine structure [23] spectroscopies. The surface presents a non-reconstructed (1 × 1) bulk termination, as shown in Fig. 1. The sawtooth corrugation of the surface is composed of five- and six-fold coordinated Ti atoms, (Ti_5c_, Ti_6c_) and two- and three-fold coordinated O atoms (O_2c_, O_3c_).

Similar to rutile (110), the O_2c_ atoms at the anatase (101) surface can be removed during sample preparation or by electron bombardment [11, 18], forming oxygen vacancies (O-vacs). The creation of O-vacs leads to a reduced surface, and induces Ti 3d band gap states (BGS) ~1 eV below the Fermi level (*E*_F_) [24, 27–29]. This points to the formation of a small polaron at the anatase (101) surface upon O-vac creation [12, 22]. Monoatomic-height step edges contribute a non-negligible fraction of surface atoms [20] and have also been shown to be associated with states in the band gap region [12].

The adsorption of water on anatase (101) is of great interest due to its presence in many of the applications of TiO_2_, and has recently been described in two review articles [6, 30]. Calculations have predicted that water dissociates at surface O-vacs, forming two bridging hydroxyls (OH) per vacancy [31]. As with rutile (110), anatase (101) remains reduced after hydroxylation and is predicted to have an associated BGS [31, 32]. The anatase (101) surface has been shown to have a higher concentration of O-vacs than rutile (110) in resonant photoemission measurements [24]. However, an interesting difference between rutile (110) and anatase (101) is the relative absence of point defects such as surface O-vacs in scanning tunnelling microscopy (STM) images [19]. Subsequent STM measurements have shown that O-vacs at the anatase (101) surface migrate to the sub-surface region at temperatures above 200 K, reaching a thermally equilibrated coverage of ~1 % of a monolayer for the as-prepared surface at room temperature [18]. Although less reactive than surface O-vacs, sub-surface O-vacs still influence water adsorption by shifting their desorption to higher temperatures, suggesting stronger binding due to these defects [33]. O-vacs have also been shown to aggregate, forming sub-surface clusters upon heating above room temperature [11]. The preference for sub-surface O-vacs on the anatase (101) surface reconciles the apparent discrepancy between STM and resonant photoemission studies.

Consequently, sample preparation under ultra-high vacuum conditions results in surfaces possessing very low concentrations of surface O-vacs [18]. The relative scarcity of these highly reactive point defects likely explains the consensus that water predominantly adsorbs molecularly on the as-prepared anatase (101) surface [7, 19, 26, 30, 31, 33, 34]. Temperature-programmed desorption (TPD) measurements of water on anatase (101) show peaks at 250, 190 and 160 K [26]. The 250 K peak was assigned to chemisorption of water at Ti_5c_ sites. The second water layer physisorbs at O_2c_ atoms at ~190 K, followed by the growth of multilayer water below 160 K. X-ray photoemission measurements indicate that all water layers adsorb molecularly [26] and dynamic simulations support this assignment [34]. However, there is also evidence of a mixed monolayer of water and OH on anatase (101) [25, 35].

As with many metal oxides, the surface chemistry and physics of TiO_2_ is heavily influenced by the presence of surface defects [36]. The creation of defects such as O-vacs, OH and Ti interstitial atoms leads to excess electrons [36, 37], which can become trapped in the crystal lattice, forming polarons [38]. In contrast to rutile (110) [39, 40], excess electrons at the anatase (101) surface can only be trapped at defects [12, 15]. Hence, defects act as charge-trapping centres, accumulating excess electrons, and are consequently often the preferred sites for adsorption [1, 19, 36]. Since the transfer of excess electrons to adsorbed molecules facilitates redox reactions at the surface, insight into the nature of defects in TiO_2_ is crucial for a complete understanding of its catalytic applications.

In this article we present ultraviolet (UPS) and two-photon (2PPE) photoemission spectroscopy measurements from the as-prepared, water-covered and electron-bombarded anatase TiO_2_(101)(1 × 1) surface. We find that water predominantly adsorbs molecularly on the as-prepared surface at low temperatures. At room temperature a low coverage of OH on the as-prepared surface is attributed primarily to dissociative desorption of water at step edges. This process saturates in the residual vacuum. Additional OH formation is possible at surface O-vacs created by electron bombardment, which also leads to an increase of the BGS peak above the thermally equilibrated level for the as-prepared surface. The additional excess electrons trapped by OH may increase the propensity for redox reactions to occur at the surface.

## Experimental

A natural anatase TiO_2_(101)(1 × 1) single crystal sample was prepared by cycles of Ar^+^ sputtering for 30 min and thermal annealing up to ~950 K for 10 min. The surface order was confirmed by LEED and the contamination level was measured by Auger electron (AES) and X-ray photoemission (XPS) spectroscopies, which evidenced the presence of <3 % of a monolayer of carbon. The minimum time allowed between annealing and photoemission measurements was 15 min. This time period is sufficient to allow surface O-vacs to migrate into the sub-surface region [18], resulting in what will be referred to below as the ‘as-prepared surface’. To create additional surface O-vacs non-thermally, the sample was irradiated with electrons from a fully out-gassed electron gun (500–550 eV, 13 *μ*Amm^−2^). Water deliberately dosed into the vacuum chamber was cleaned with repeated freeze–pump–thaw cycles.

Measurements employed an instrument (base pressure ~4 × 10^−10^ mbar) and laser system described elsewhere [41]. 2PPE (*hν* = 3.10–3.54 eV, 400–350 nm, 0.3–0.5 mW, spot diameter ~0.5 mm) and UPS (He-I, *hν* = 21.2 eV, 58 nm and He-II, *hν* = 40.8 eV, 30 nm) spectra were recorded using a pass energy of 10 eV. A bias voltage of −3 V was applied to the sample during the acquisition of 2PPE spectra. All photoemission spectra were acquired at room temperature, unless otherwise stated. Calibration of the sample *E*_F_ was made in reference to that of the Ta sample holder, which was also measured via photoemission.

## Results

### Water Adsorption at 130 K

**Fig. 2:**
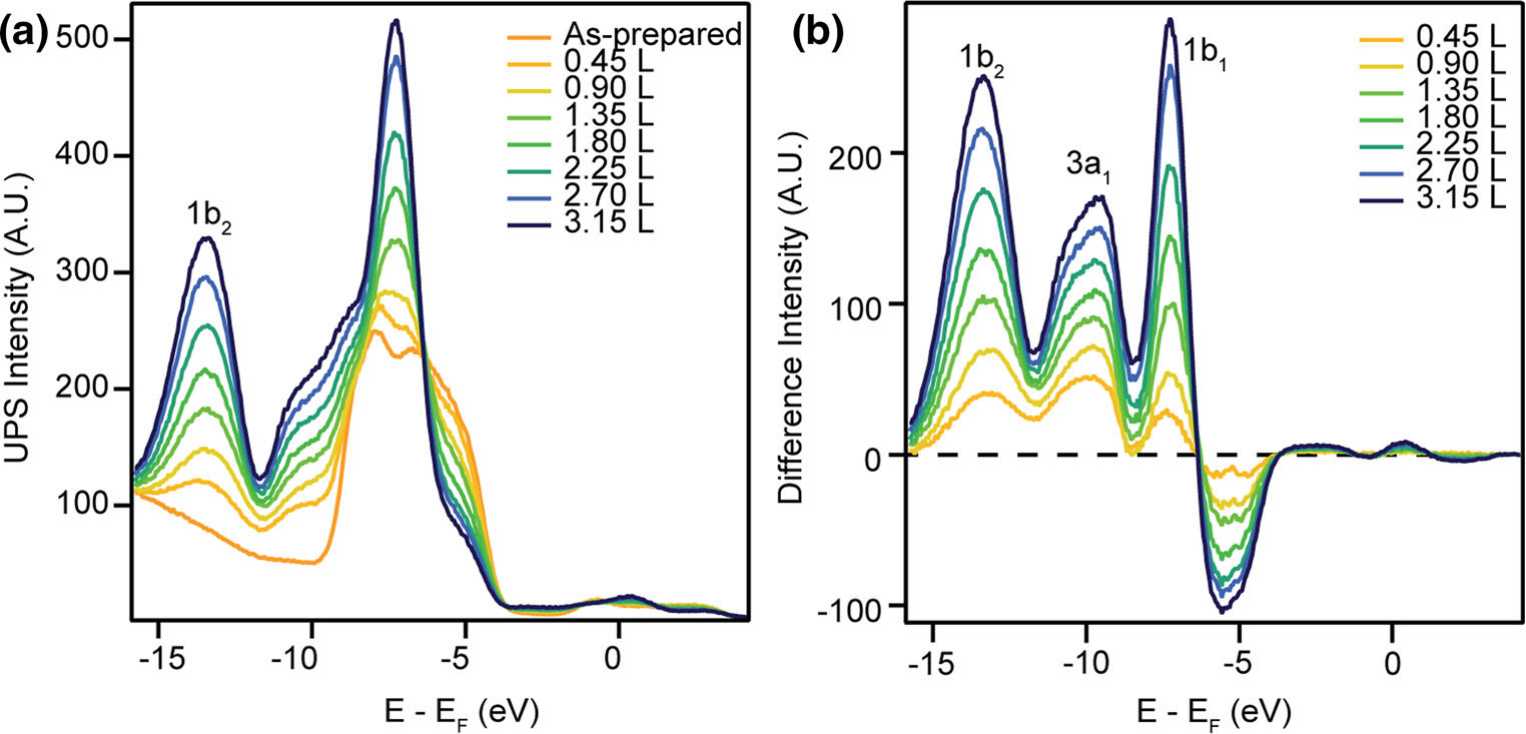
**a** UPS He-II (*hν* = 40.8 eV) spectra from the as-prepared and water-covered anatase (101) surface at ~130 K. **b** Difference spectra obtained by subtraction of the as-prepared-surface spectrum from those measured from the water-covered surface at different exposures. Three peaks associated with the molecular orbitals of water are seen at all coverages of water, labelled as 1b_2_, 3a_1_ and 1b_1_

The as-prepared surface was cooled to ~130 K before exposure to water in steps of 0.45 L (1 L = 1.33 × 10^−6^ mbar.s) up to a total exposure of 3.15 L, above which no alteration was seen in the spectra. 2PPE measurements showed the maximum change in the sample workfunction to be ~−0.9 eV, similar to the value previously measured via photoemission from water-covered rutile (110) [42–44]. UPS He-II spectra recorded after each exposure to water, and subsequently normalised to the photoelectron background, are shown in Fig. 2a. Changes in the valence band region and the growth of a feature centred at ~−13 eV are seen for all water exposures.

Figure 2b shows difference spectra obtained by subtracting the spectrum measured from the as-prepared surface from those after water exposure. The difference spectra exhibit three peaks at all water exposures, centred at 13.16, 9.91 and 7.33 eV below *E*_F_. We associate these peaks with the 1b_2_, 3a_1_ and 1b_1_ molecular orbitals of water. This assignment is made by comparison to photoemission measurements of molecular water on rutile TiO_2_(110) and in the gas phase [45, 46]. Adsorbed water hybridises with the sample’s valence band, leading to a redistribution of the O 2p levels [32, 46]. Depopulation of these levels may explain the peak with negative intensity around −5.5 eV in Fig. 2b. The peaks show no significant change in energy with water exposure; however, the 3a_1_ molecular orbital peak appears asymmetrical in all but the 0.45 L difference spectrum. It is known that shifts in the 3a_1_ molecular orbital peak can arise from hydrogen bonding and other adsorbate–adsorbate interactions, which may explain this variation with water coverage [47].

### Electron Bombardment

**Fig. 3:**
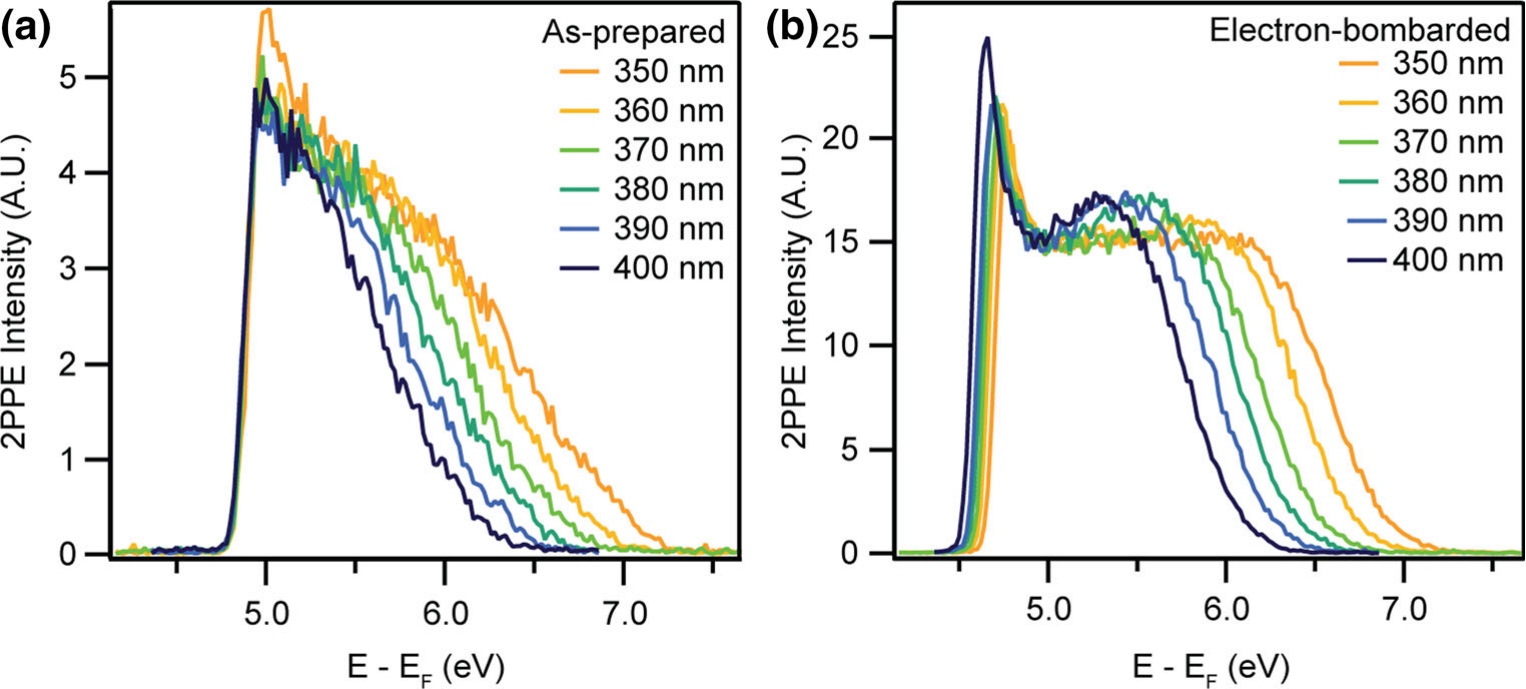
**a** 2PPE (*hν* = 3.10-3.54 eV) spectra from the as-prepared anatase (101) surface. The sample workfunction is 4.8 eV. The spectra are normalised to the intensity at 5.2 eV. **b** 2PPE spectra after irradiation with 500 eV electrons for 2 min. The workfunction is reduced by ~0.2 eV and a new peak appears ~5.5–6.5 eV above E_F_, in comparison to the as-prepared surface. The spectra are normalised to the intensity at ~5.0 eV

Electron bombardment of TiO_2_ is known to create point defects such as O-vacs at the surface non-thermally [11, 18]. The as-prepared anatase surface was bombarded with 500 eV electrons for 2 or 5 min before 2PPE and UPS spectra were measured. 2PPE spectra of the as-prepared surface obtained using photons between 400 nm (3.10 eV) and 350 nm (3.54 eV) are shown in Fig. 3a. Since photons below ~320 nm (above 3.9 eV) are required to stimulate 2PPE from the valence band, photoemitted electrons in Fig. 3 must originate from the BGS. The workfunction of the as-prepared surface was 4.8 eV, similar to the value of (4.7 ± 0.1) eV previously reported for the anatase single crystal TiO_2_(101) surface [48].

**Fig. 4:**
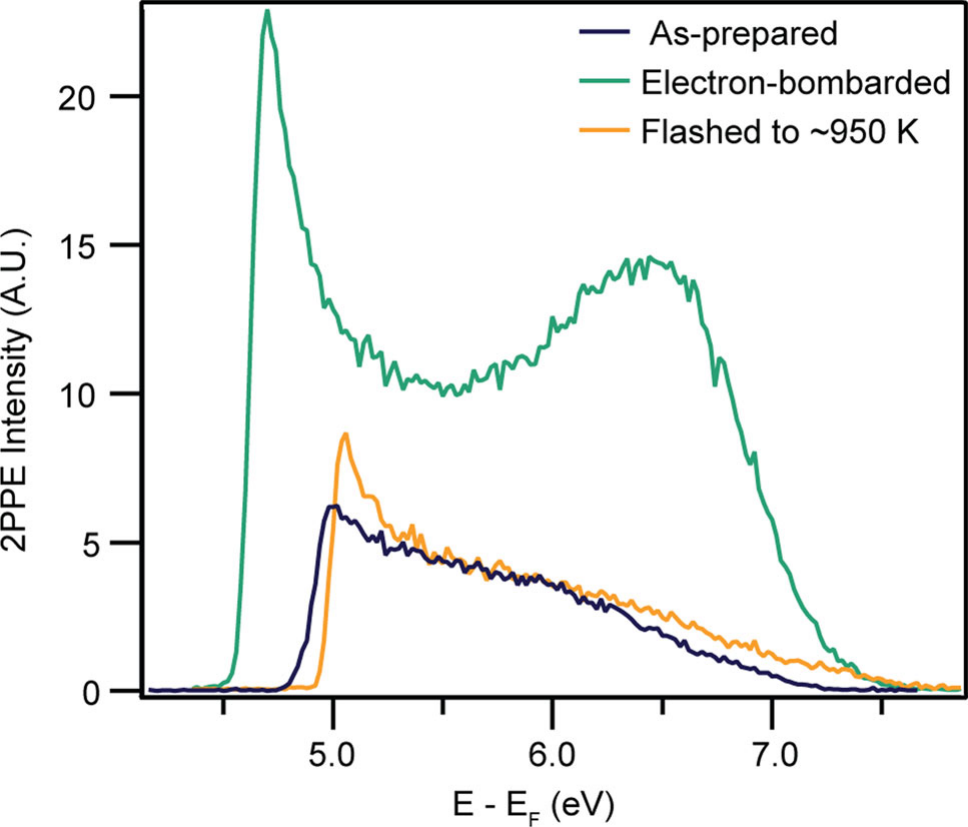
2PPE spectra (*hν* = 3.54 eV) from the as-prepared, electron-bombarded (500 eV, 2 min) and flashed anatase (101) surface. The increase in the feature ~6.5 eV above *E*_F_ in the electron-bombarded spectrum relative to that from as-prepared surface suggests that the sample is more reduced. Flashing the sample to ~950 K results in a spectrum most similar to that from the as-prepared surface, suggesting that the original surface defect concentration has been recovered

2PPE spectra measured following bombardment with 500 eV electrons for 2 min are shown in Fig. 3b. These spectra show three main differences from those of the as-prepared surface. Firstly, the sample workfunction is reduced by ~0.2 eV. Quantifying this change is complicated by the appearance of peaks at the workfunction cut-off attributed to space-charge effects. Secondly, a feature appears between ~5.5–6.5 eV above *E*_F_, depending upon the photon energy. Finally, the 2PPE intensity is much higher from the electron-bombarded surface, as seen from the spectra in Fig. 4, which have not been normalised. The effect of beam damage by the laser on the bombarded region can be excluded, as no change occurred in the 2PPE spectra after irradiation with a high flux of 350 nm photons (0.2 mJcm^−2^ per pulse) for 60 min, compared to the flux used during measurements (0.04 mJcm^−2^ per pulse). Upon flashing the sample to ~950 K, a 2PPE spectrum closely resembling that of the clean surface is recovered, as seen in Fig. 4. This suggests that the heating process heals the effects of electron bombardment on the surface.

**Fig. 5:**
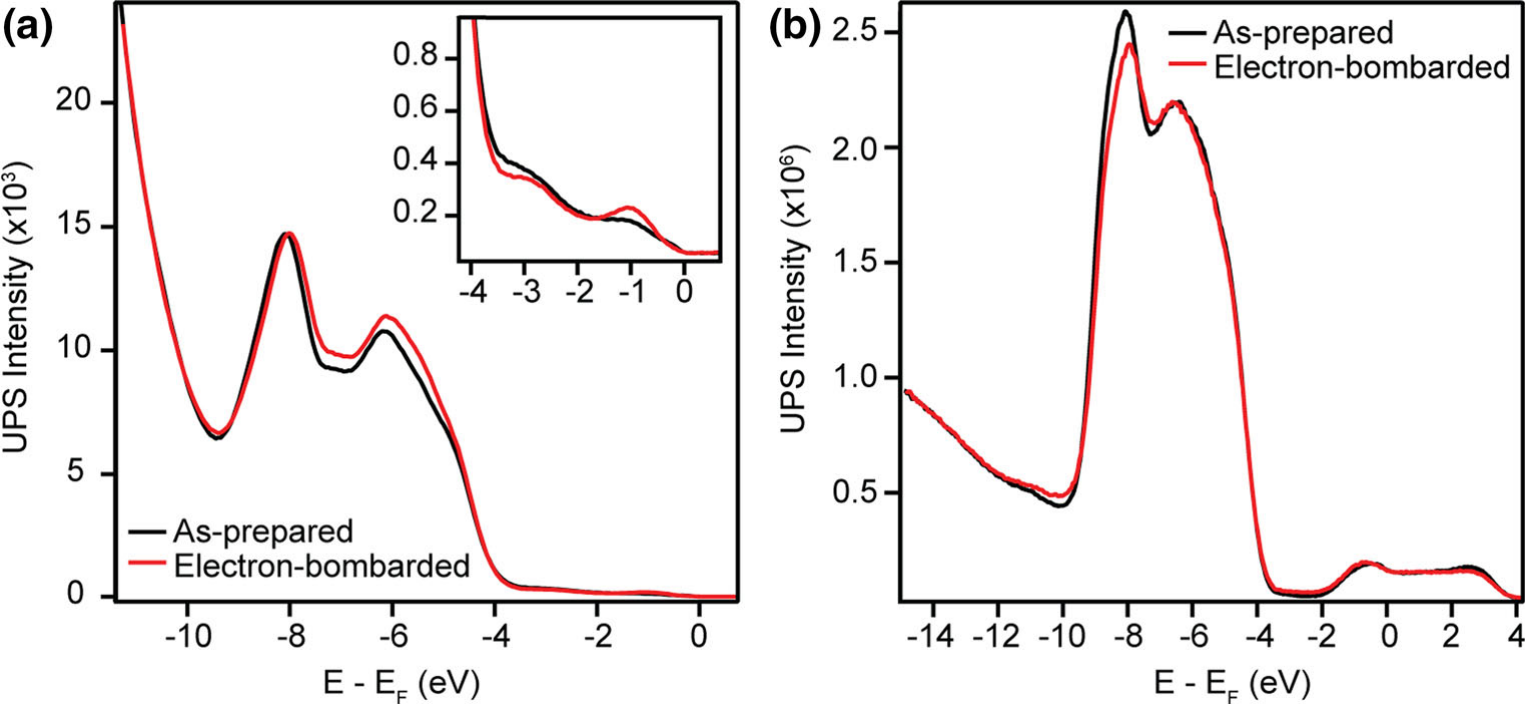
**a** UPS He-I (*hν* = 21.2 eV) spectra from the as-prepared and electron-bombarded (500 eV, 2 min) anatase (101) surfaces. Irradiation of the surface induces changes in the valence band region and an increase of the BGS peak (shown in the *inset*). **b** UPS He-II (*hν* = 40.8 eV) spectra from the as-prepared and electron-bombarded anatase (101) surfaces showing irradiation induced changes in the valence band region

UPS He-I measurements from the as-prepared and electron-bombarded surfaces are shown in Fig. 5a, normalised to the photoelectron background. Electron bombardment of the surface increased the photoemission signal relative to the as-prepared surface, at energies approximately 9.4 eV, 7.0–4.5 and 0.7 eV below *E*_F_. The He-I spectra were fitted in the region 3.4 eV below to 0.4 eV above *E*_F_, as described in Ref. [44]. The BGS peak appears (0.75 ± 0.05) eV below *E*_F_, in agreement with previous measurements [12, 22], with a peak area enhanced by a factor of 1.50 following electron bombardment.

Figure 5b shows He-II spectra from the same surface, normalised to the photoelectron background. The spectra reveal new features around 10.5 and 7.2 eV below *E*_F_ following electron bombardment. The photoemission signal in the region of −2 to 4 eV arises principally from valence band emission from the He-II β emission line (*hν* = 48.4 eV) and is therefore not considered. A shift of ~0.05 eV was made to both the He-I and He-II spectra of the electron-bombarded surface to correct for band banding away from *E*_F_, which is induced by the reduction of the surface. Modifications to the valence band via electron bombardment may also influence the background of inelastically scattered electrons. These scattered electrons contribute significantly higher intensity to He-I than He-II spectra. Hence, direct comparisons between the He-I and He-II spectra in Fig. 5 are avoided in this manuscript.

**Fig. 6:**
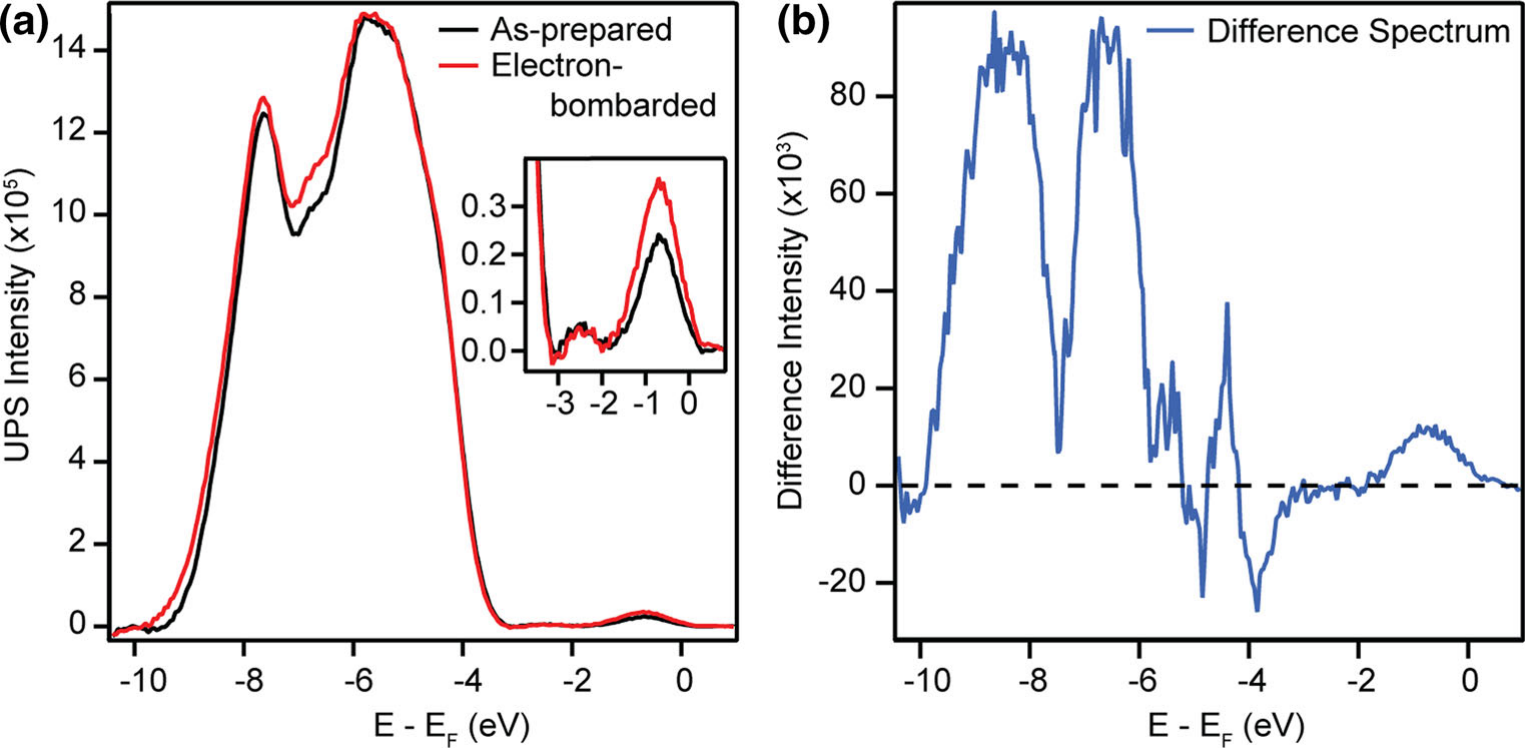
**a** UPS He-I (*hν* = 21.2 eV) spectra from the as-prepared and electron-bombarded (500 eV, 5 min) anatase (101) surfaces following the subtraction of a polynomial background. Irradiation-induced changes in the valence band region and an increase in the BGS peak (shown in the *inset*) are evident. **b** Difference spectrum obtained by subtracting the as-prepared spectrum from the electron-bombarded spectrum in (**a**). Two main peaks are evident, centred 8.45 and 6.50 eV below *E*_F_. The feature ~4.4 eV below *E*_F_ possibly arises from a change in gradient of the valence band maximum. The peak centred ~0.7 eV below *E*_F_ arises from irradiation-induced enhancement of the BGS peak due to the creation of O-vacs

In order to increase the magnitude of the BGS enhancement and changes in the valence band region, a clean anatase (101) sample was irradiated with 500 eV electrons for a longer time of 5 min. The resulting He-I spectra are shown in Fig. 6a, following normalisation and subtraction of a fifth order polynomial background. Excess electrons trapped at step edges create states in the band gap of anatase, whose energy distribution closely resembles the background present in photoemission spectra [12]. Contributions from these states may be removed when subtracting a polynomial background from our UPS spectra. However, since electron bombardment is expected primarily to create point defects at the surface rather than step edges, it should not significantly alter the density of excess electrons trapped at step edges. Similar to the spectra in Fig. 5, the BGS peak area in Fig. 6a was seen to increase by a factor of 1.53. Hence, increasing the irradiation time from 2 to 5 min did not significantly increase the enhancement of the BGS peak area.

To see the effect of electron bombardment on the He-I spectra in Fig. 6a more clearly, the as-prepared spectrum was subtracted from the electron-bombarded spectrum. The resulting difference spectrum, shown in Fig. 6b, reveals two main changes to the He-I spectra following electron bombardment, which are centred 8.45 and 6.50 eV below *E*_F_. These peaks are associated with the 3σ and 1π molecular orbitals of OH, respectively (see below). Additionally, a smaller peak is present 4.4 eV below *E*_F_, which may originate from a change in the gradient of the valence band maximum. However, difference spectra are highly sensitive to small changes in intense bands and although care was taken to correct for this, the feature may arise from a slight misalignment of the spectra. The feature ~0.75 eV below *E*_F_ originates from enhancement of the BGS peak.

### Electron Bombardment with Increased Water Exposure

Finally, the as-prepared anatase (101) sample was irradiated by 550 eV electrons for 3.5 min, with and without simultaneous exposure to (6.6 × 10^−8^) mbar H_2_O (~10.5 L) at room temperature. Mass spectra measured during exposure of the surface to water revealed that oxygen was also present at a partial pressure of <(3 × 10^−9^) mbar (~0.5 L). The residual pressure of water without deliberate dosing was estimated to be less than (2 × 10^−10^) mbar.

**Fig. 7:**
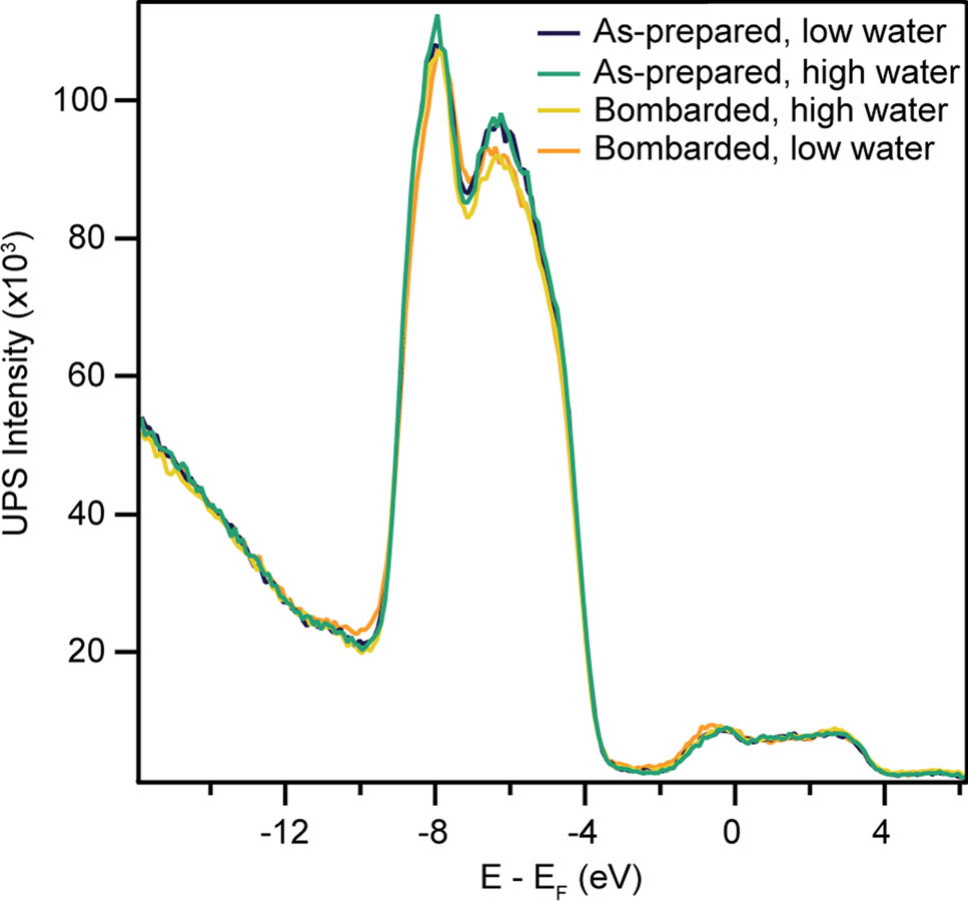
UPS He-II (*hν* = 40.8 eV) spectra from the as-prepared and electron-bombarded (550 eV, 3.5 min) anatase (101) surfaces at high (6.6 × 10^−8^ mbar) and low (<2× 10^−10^ mbar) water pressures. Only irradiation at low water pressures resulted in changes in the spectra in comparison to those from the as-prepared surface. The absence of changes at high water pressure may be explained by contamination of the water by oxygen, which heals the surface O-vacs created via electron bombardment

Figure 7 displays He-II spectra measured from the as-prepared and electron-bombarded surfaces with and without water exposure. A small feature exists in spectra from the as-prepared surface with and without water exposure, which appears ~11 eV below *E*_F_ and is attributed primarily to dissociative water adsorption at step edges. The spectrum from the surface electron-bombarded at low water pressure exhibits a shoulder about ~10 eV below *E*_F_, which is not present in the other spectra. The similarity of the spectrum from the surface electron-bombarded with high water pressure to the as-prepared spectra suggests that this process did not alter the surface. Additionally, the BGS peak area in He-I spectra was not affected by electron bombardment during exposure to 10.5 L H_2_O.

## Discussion

Despite receiving considerable attention, the adsorption behaviour of water on TiO_2_ surfaces remains controversial. In Fig. 2, adsorption of water at ~130 K was shown to create three peaks in He-II difference spectra which are associated with the molecular orbitals of water. The position of the 3a_1_ molecular orbital peak is shifted 0.5 eV away from *E*_F_ on anatase (101) compared to that of gas phase water. This is similar to the 0.4 eV stabilisation previously measured on rutile (110) [45, 46]. The 3a_1_ and 1b_1_ molecular orbitals of water overlap and hybridise with the sample’s valence band upon adsorption [32, 49], complicating comparison with calculations. However, the position of the 1b_2_ molecular orbital peak agrees well with recent calculations [32].

We find little evidence for dissociative water adsorption at low temperatures in He-II measurements. This finding is in agreement with many experimental and theoretical works, which suggest water adsorbs molecularly on the defect-free anatase (101) surface [7, 19, 21, 26, 31, 33, 34, 50, 51]. It is possible however, that a mixed monolayer of water exists on anatase (101) at higher temperatures, as suggested in Ref. [25] and [35]. Additionally, a small feature is present in He-II spectra of the as-prepared surface acquired at room temperature, ~11 eV below *E*_F_ which we attribute to dissociative adsorption of water at step edges.

Enhancement of the BGS peak area in He-I spectra by a factor of ~1.5 was observed following electron bombardment for both 2 and 5 minutes. Additionally, 2PPE measurements support an increase in the reduction level of the sample following electron bombardment. These results suggest that it is possible to trap additional excess electrons at the anatase (101) surface above the thermally equilibrated concentration via electron bombardment at room temperature. 2PPE spectra reveal that the equilibrium concentration is recovered upon heating the sample to ~950 K.

It is known from STM studies that electron bombardment creates O-vacs on the anatase (101) surface [11, 18]. The creation of O-vacs further reduces the surface, which is expected to induce band bending away from *E*_F_ and increase the BGS peak intensity. Both these changes were observed in our UPS measurements, in addition to two new features in the valence band region. O-vac creation is known to alter the appearance of the valence band in photoemission spectra. A feature 6.7 eV below *E*_F_ in resonant photoemission spectra, similar to that at −6.50 eV in Fig. 6b, was attributed to hybridisation between the Ti 3d t_2g_ or 4sp orbitals and the O 2p orbitals [24]. However, this feature was attenuated by the reduction of the surface whereas the feature at −6.50 eV in Fig. 6b increases as the surface becomes more reduced. Hence, we suggest that other contributions may exist in this energy range.

We propose that the peaks centred 8.45 and 6.50 eV below *E*_F_ in Fig. 6b are associated with the 3σ and 1π molecular orbitals of OH, respectively. This OH may be created by dissociative adsorption of water in the residual vacuum at surface O-vacs created during electron bombardment. Theoretical work predict that this process results in two bridging OH per surface oxygen vacancy [31, 51]. Indeed, the position of the OH 3σ molecular orbital peak in our measurements is in good agreement with DFT calculations [49].

In photoemission spectra of the hydroxylated rutile (110) surface, the OH 3σ and 1π molecular orbitals peaks appear 10.8–10.2 and 8.0–7.6 eV below *E*_F_, respectively [45, 46]. The ~2 eV separation between the OH molecular orbital peaks in Fig. 6b agrees particularly well with that seen for hydroxylated rutile (110) in Ref. [45]. Note also that the peak ~11 eV below *E*_F_ in Fig. 7, assigned tentatively to OH formation at step edges, will not contribute intensity to difference spectra as this feature is already present in spectra from the as-prepared surface.

In comparison to hydroxylated rutile (110), we find that the molecular orbitals of OH on anatase (101) are shifted ~1.5 eV towards *E*_F_. The increased proximity of states associated with the OH 1π molecular orbital to the valence band maximum may contribute to the increased catalytic activity of anatase over rutile TiO_2_, as this increases the localisation of the highest occupied molecular orbital and therefore its ability to trap holes [32]. Since the OH 1π molecular orbital overlaps with the valence band, it is expected to hybridise significantly with the surface electronic structure upon adsorption [32, 49].

The creation of OH at the anatase (101) surface also explains the enhancement of the BGS peak in He-I spectra following electron bombardment. Since excess electrons at the anatase (101) surface can only be trapped by defects [12, 15], dissociative adsorption of water at surface O-vacs may fix these excess electrons to the surface and prevent defect migration into the sub-surface region or bulk. Recent DFT calculations have shown the nature of polarons at surfaces of rutile TiO2 and other metal oxides to be independent of their donor defect [52]. Hence, it is likely that excess electrons at the anatase TiO_2_(101) surface are localised in the vicinity of the OH, as has been observed for surface O-vacs [15]. Subsequently, additional excess electrons trapped at OH may increase the potential for redox reactions to occur at the surface, compared to the as-prepared surface. Heating the sample to ~950 K is expected to cause desorption of OH. Indeed, the 2PPE spectrum measured after heating the sample appears similar to that of the thermally equilibrated, as-prepared surface.

Finally, the surface was electron-bombarded during exposure to a higher partial pressure of water, resulting in He-I and He-II spectra similar to those from the as-prepared surface. Hence, it appears that this process heals the majority of defects created during electron bombardment. Since the adsorption of neutral water is not expected to alter the reduction level of the surface, the most likely explanation arises from the ~5 % oxygen contamination seen in mass spectroscopy measurements.

## Conclusion

The effects of water adsorption and electron bombardment on the anatase TiO_2_(101)(1 × 1) surface have been studied via 2PPE and UPS. On the as-prepared surface, He-I measurements suggest that water primarily adsorbs molecularly at low temperatures, probably due to a near-negligible coverage of surface O-vacs. However, thermal effects may also play a role in preventing dissociative adsorption. Electron bombardment of the as-prepared surface at room temperature was found to increase the BGS peak intensity by a factor of 1.5. This enhancement above the thermally equilibrated level was explained by the dissociative adsorption of water from the residual vacuum at surface O-vacs, resulting in two bridging OH per vacancy [31, 51]. Peaks associated with the OH 3σ and 1π molecular orbitals were assigned to new features in the photoemission spectra centred 8.45 and 6.50 eV below *E*_F_, respectively. The ability to increase the concentration of excess electrons at the surface of anatase (101) may increase the potential for redox reactions to occur at the surface.
